# Long-Read Genome Sequence of the Sugar Beet Rhizosphere Mycoparasite *Pythium oligandrum*

**DOI:** 10.1534/g3.119.400746

**Published:** 2019-12-02

**Authors:** Charlène Faure, Marine Veyssière, Betty Boëlle, Hélène San Clemente, Olivier Bouchez, Céline Lopez-Roques, Adeline Chaubet, Yves Martinez, Karel Bezouška, Martin Suchánek, Elodie Gaulin, Thomas Rey, Bernard Dumas

**Affiliations:** * Laboratoire de Recherche en Sciences Végétales, Université de Toulouse, CNRS, UPS, 24 Chemin de Borde Rouge, Auzeville, BP42617, 31326 Castanet-Tolosan, France,; † INRA, US 1426, GeT-PlaGe, Genotoul, Castanet-Tolosan, France,; ‡ CNRS, Fédération de Recherches Agrobiosciences, Interactions, Biodiversity, Plateforme d’Imagerie TRI, Castanet-Tolosan, France,; § Biopreparáty spol. s r. o., Tylišovská 772/1, 16000 Praha 6, Czech Republic, and; ** De Sangosse, Bonnel, 47480, Pont-Du-Casse, France

**Keywords:** *Pythium oligandrum*, genome, PacBio, RNAseq, Mycoparasitism

## Abstract

*Pythium oligandrum* is a soil born free living oomycete able to parasitize fungi and oomycetes prey, including important plant and animals pathogens. *Pythium oligandrum* can colonize endophytically the root tissues of diverse plants where it induces plant defenses. Here we report the first long-read genome sequencing of a *P. oligandrum* strain sequenced by PacBio technology. Sequencing of genomic DNA loaded onto six SMRT cells permitted the acquisition of 913,728 total reads resulting in 112X genome coverage. The assembly and polishing of the genome sequence yielded180 contigs (N50 = 1.3 Mb; L50 = 12). The size of the genome assembly is 41.9 Mb with a longest contig of 2.7 Mb and 15,007 predicted protein-coding genes among which 95.25% were supported by RNAseq data, thus constituting a new *Pythium* genome reference. This data will facilitate genomic comparisons of *Pythium* species that are commensal, beneficial or pathogenic on plant, or parasitic on fungi and oomycete to identify key genetic determinants underpinning their diverse lifestyles. In addition comparison with plant pathogenic or zoopathogenic species will illuminate genomic adaptations for pathogenesis toward widely diverse hosts.

Oomycetes are filamentous or unicellular ubiquitous eukaryotic organisms. For many years, oomycetes were considered to be fungi because of their ecological and morphological similar traits. However, they are distinct from the fungal group and phylogenetically belong to the SAR (stramenopila – alveolata – rhizaria) supergroup. Oomycetes are divided into three major groups: Basal, Saprolegnialean and Peronosporalean. They are mostly known because of their pathogenic representatives able to parasite algae, mammals, aquatic animals, plants, and filamentous fungi (Thines 2018). Knowledge on the genomes of oomycetes is dominated by studies carried out on devastating plant pathogens such as *Phytophthora* spp. (Haas *et al.* 2009). The *Pythium* genus belongs to the *Pythiaceae* family, in the Pythiales order, in the Peronosporalean lineage from the class Oomycota (Uzuhashi *et al.* 2010). This genus is widespread both in water and in soil and harbors species with contrasting lifestyles. Almost 150 species have already been described and many of them are known to be plant pathogens such as *P. ultimum* (Martin and Loper 1999). Phytopathogenic *Pythium* spp. cause in most cases root and stem rots and fruit and damping-off of seeds and seedlings. Other species are known to be pathogenic on animals including mammals (*P. insidiosum,(**Gaastra et al. 2010**)* or insects (*P. guiyangense*, Shen *et al.* 2019). In addition, this genus includes several mycoparasitic species, notably *P. oligandrum* or *P. periplocum (*Ribeiro and Butler 1995*)* which are able to parasitize fungi and oomycetes by developing specialized infection structures and ultimately killing their prey (Figure 1). These different lifestyles lean on the abilities of Pythium spp. to degrade widely diverse host cell walls. Hence, their Carbohydrate Degrading enzymes toolkit, often referred as CAZyomes may be relevant to explain the diversity of *Pythium* lifestyle and host range (Zerillo *et al.* 2013; Zhang *et al.* 2018).

**Figure 1:**
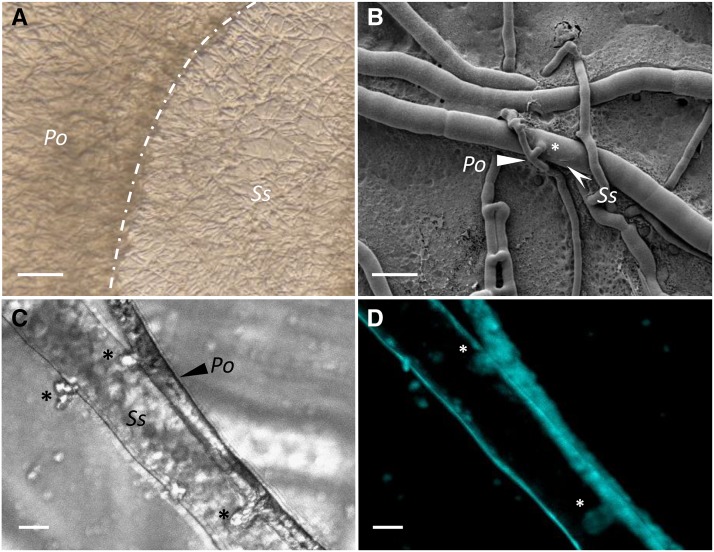
*Pythium oligandrum* mycoparasitic structures on the phytopathogenic fungus *Sclerotinia sclerotiorum*. (A) Observation of confrontation zone (dotted line) between *Pythium oligandrum (Po)* and *Sclerotinia sclerotiorum* (*Ss)* at 3 days post inoculation using pseudo Differential Interference Contrast (DIC) microscopy (scale bar = 200 µm). (B) Appressorium-like structures (asterics,*) developed by *P. oligandrum* on *S. scleotiorum* hyphae are visible under a Scanning Electron Microscope (SEM), (scale bar = 25 µm). (C) *P. oligandrum* hyphae projecting lateral hyphae inside *S.sclerotiorum* cell (asterics,*) monitored using a Transmitted light microscope and (D) cell walls calcofluor stain signals of a confocal maximum projection (scale bar = 5 µm).

*Pythium oligandrum* is also a commensal or beneficial colonizer of plant roots (Le Floch *et al.* 2005), thus this oomycete species has a huge biotechnological relevance for agriculture as biological control agent (Benhamou *et al.* 2012).

*Pythium oligandrum* ATCC 38472 was isolated by Dáša Veselý in 1972 (Veselý 1977) as an indigenous wild type strain from sugar beet-associated soil near Semčice in the former Czechoslovakia (now the Czech Republic). In 1979 it was deposited in ATCC under the number 38472, then, in the 1980s it was first used by the Slušovice cooperative in the agricultural product against damping-off of sugar beet. In the 1990s, after the cessation of Slušovice cooperative, Dáša Veselý licensed the strain exclusively to the company Biopreparáty for the production of the biological fungicide *Polyversum*.

Two other *P. oligandrum* strains were previously sequenced by Illumina technology, Po37 (Berger *et al.* 2016) (Genbank LSAJ 01000000) and CBS 530.74 (Kushwaha *et al.* 2017) (Genbank NAJK00000000). However, our ATCC 38472 sequencing project is the first long-read genome of a *P. oligandrum* strain. We expect that this reference genome will be used for genomic comparison of plant beneficial or commensal *Pythium spp*. such as ATCC 38472 with other mycoparasitic *Pythium* spp. to foster the identification of key genetic determinants shaping this lifestyle whereas comparison with plant pathogenic or zoopathogenic species will illuminate Pythiales genomic adaptations for pathogenesis toward widely diverse hosts.

## Materials and Methods

### Pythium oligandrum strain and mycoparasitism assay on Sclerotinia sclerotiorum

An isolate of *P. oligandrum* Drechsler ATCC 38472 was recovered from the commercial product Polyversum. Observations of mycoparasitism events observations were obtained using non fluorescence optical microscopy (Stereomicroscope Nikon SMZ 18), confocal microscopy (Leica TCS SP8) and scanning electron microscopy (FEG FEI Quanta 250). Hyphal interactions between *P. oligandrum* and *S. sclerotiorum* were studied according the following procedure. A solution of Polyversum (5 mg/ml) was set up and 200 µL of the solution was spread on a Potato Dextrose Agar (PDA) medium Petri dish, and dried under the hood for 5 min. Agar plugs (5 mm in diameter) were collected from the growing margin of fresh cultures of *S. sclerotinia* and placed at the center of the Petri dish containing the Polyversum and grown 3 days at 22° before microscopical analysis. For confocal microscopy, observations were made following tissues staining with Calcofluor White (concentration 100 µg/ml, incubation time 30 min, two washes, remove in water’) enabling staining of both fungal and oomycete derived cell walls.

### DNA extraction for long-read sequencing and library preparation

The oomycete was cultivated in liquid Potato Dextrose Broth (PDB) medium at 28° for 48h. Approximately 300 mg of fresh mycelium ground with liquid nitrogen and genomic DNA was isolated using the Macherey-Nagel Nucleobond RNA/DNA kit according to the manufacturer’s instructions. Library preparation and sequencing were performed at the GeT-PlaGe core facility, INRA Toulouse, according to the manufacturer’s instructions “Shared protocol-20kb Template Preparation Using BluePippin Size Selection system (15kb size Cutoff)”. At each step, DNA was quantified using the Qubit dsDNA HS Assay Kit (Life Technologies). DNA purity was tested using the NanoDrop (Thermo Fisher) and size distribution and degradation assessed using High Sensitivity Large Fragment 50kb Analysis Kit used with a Fragment analyzer (AATI). Purification steps were performed using 0.45X AMPure PB beads (PacBio). A total of 10 µg of DNA was purified then sheared at 40kb using the megaruptor system (Diagenode).

### SMRT sequencing and De novo assembly

Using SMRTBell template Prep Kit 1.0 (PacBio), a DNA and END damage repair step was performed on 5 µg of sample. Then, blunt hairpin adapters were ligated to the library. The library was treated with an exonuclease cocktail to digest unligated DNA fragments. A size selection step using a 10kb cutoff was performed on the BluePippin Size Selection system (Sage Science) with 0.75% agarose cassettes, Marker S1 high Pass 15-20 kb. Conditioned Sequencing Primer V2 was annealed to the size-selected SMRTbell. The annealed library was then bound to the P6-C4 polymerase using a ratio of polymerase to SMRTbell at 10:1. Then after a magnetic bead-loading step (OCPW), SMRTbell libraries were sequenced on 6 SMRTcells on RSII instrument at 0.18 to 0.23 nM with a 360 min movie. The initially generated raw sequencing reads were evaluated in terms of the average quality score at each position, GC content distribution, quality distribution, base composition, and other metrics. Sequencing reads with low quality were also filtered out before the genome assembly and annotation of gene structure. Finally, microbial DNA potential contamination was excluded after comparison by blast of the draft assembly of the first SMRT cell against a 16S ribosomal RNA sequences data bank (Bacteria and Archaea). The subreads were assembled with the Pacific Biosciences software SMRTanalysis version 2.3.0 using default settings with a minimum subreads length of 3 kb to exclude smaller sequenced reads and a read score of better than 0.8 to enrich in reads with a low error rate.

### RNA extraction and construction of sequencing libraries

The oomycete was cultivated on Potato Dextrose Agar (PDA) medium at 22° for 48h. Total RNA was isolated using the RNeasy Plant Mini Kit Qiagen according to manufacturer’s instructions. Three replicates were prepared for the libraries construction, each one was a pool of two PDA cultures. RNAseq was performed at the GeT-PlaGe core facility, INRA Toulouse, France. RNA-seq libraries have been prepared according to Illumina’s protocols using the Illumina TruSeq Stranded mRNA sample prep kit to analyze mRNA. Briefly, mRNAs were selected using poly-T beads. Then, RNAs were fragmented to generate double stranded cDNA and adaptators were ligated to be sequenced. Eleven cycles of PCR were applied to amplify libraries. Library quality was assessed using a Fragment Analyzer and libraries were quantified by QPCR using the Kapa Library Quantification Kit. RNA-seq experiments have been performed on an Illumina HiSeq3000 using a paired-end read length of 2x150 bp with the Illumina HiSeq3000 sequencing kits.

### Data availability

This Whole Genome Shotgun project has been deposited at DDBJ/ENA/GenBank under the accession PRJNA528861. The RNA-Seq consisting of 3 samples has been deposited in NCBI; raw reads were stored as SRA format under the accession SRR8923103. Supplemental materials are uploaded to figshare. Table S1 lists *P. oligandrum* 15,007 predicted proteins and their annotation. Table S2 lists *P. oligandrum* 1,617 predicted secreted proteins and their annotations. Table S3 is the REVIGO analysis table of the GO term occurrence related to the biological process axe of the putative secreted proteins. Table S4 is the REVIGO analysis table of the GO term occurrence related to the molecular function axe of the putative secreted proteins. Table S5 lists the 490 predicted proteins with annotated CAZY domain. Table S6 lists *P. oligandrum* 156 predicted secreted proteins with a peptidase GO annotation. Table S7 lists *P. oligandrum* 303 putative Small Secreted Proteins (SSP) with N < 900 base pairs. Table S8 lists *P. oligandrum* 27 putative Crinkler (CRN) effectors. Supplemental material available at figshare: https://doi.org/10.25387/g3.9879566.

## Results and Discussion

### Genome assembly

The sequencing of six SMRT cells allowed the acquisition of 913,728 total reads with genome coverage of 112X. The assembly and polishing of the genome using SMRT analysis yielded180 contigs (N50 = 1.3 Mb; N80 = 6.7 Kb; L50 = 12; L80 = 25). The assembly genome size is 41.9 Mb with a longest contig of 2.7 Mb. The GC percent is equal to 53% (Table 1). Thirty-three out of 180 contigs have terminal inverted repeats that may be telomeres, with a seven nucleotide repeat (5′ –TTTAGGG- 3′) (Li *et al.* 2009; Muraki *et al.* 2012). Six contigs were flanked by such sequences on both ends suggesting they correspond to full chromosome sequence. However, further studies are required to elucidate ploidy and karyotype of the strain ATCC 38472.

**Table 1:** Assembly summary

Assembly results	
Number of contigs	180
Read coverage	112X
Total contigs length / Genome size (bp)	41,970,768
Mean contig size (bp)	233,170.93
Median contig size (bp)	28,794
Longest contig (bp)	269,7281
Shortest contig (bp)	1,893
Contigs > 500 bp	180 (100.00%)
Contigs > 1K bp	180 (100.00%)
Contigs > 10K bp	162 (90.00%)
Contigs > 100K bp	37 (20.56%)
Contigs > 1M bp	16 (8.89%)
N50 (bp)	1,292,773
L50	12
N80 (bp)	672,178
L80	25

### Genome annotation

We performed a RNAseq based annotation to predict protein-coding genes using Augustus version 2.6.1 (Stanke and Morgenstern 2005) and 15,007 protein-coding gene were predicted, among which 95,25% showed expression evidence in RNAseq data. Sequencing completeness was estimated using BUSCO version 3 (Simão *et al.* 2015) based on a set of 234 common stramenopile genes, aka Benchmarking Universal Single-Copy Orthologs (BUSCOs). A total of 227 complete single-copy BUSCOs and 5 duplicated BUSCOs were found, leading to two missing BUSCOs in *P. oligandrum* ATCC 38472 which suggest a complete genome sequence assembly. The predicted proteome was functionally annotated using the InterProScan software version 4.8 and KEGG pathway. A total of 10,657 (71%) predicted proteins were successfully annotated with a domain InterPro (Table S1) while 3,912 (26%) show positive hits in KEGG (Table S1).

### Secretome

**Figure 2:**
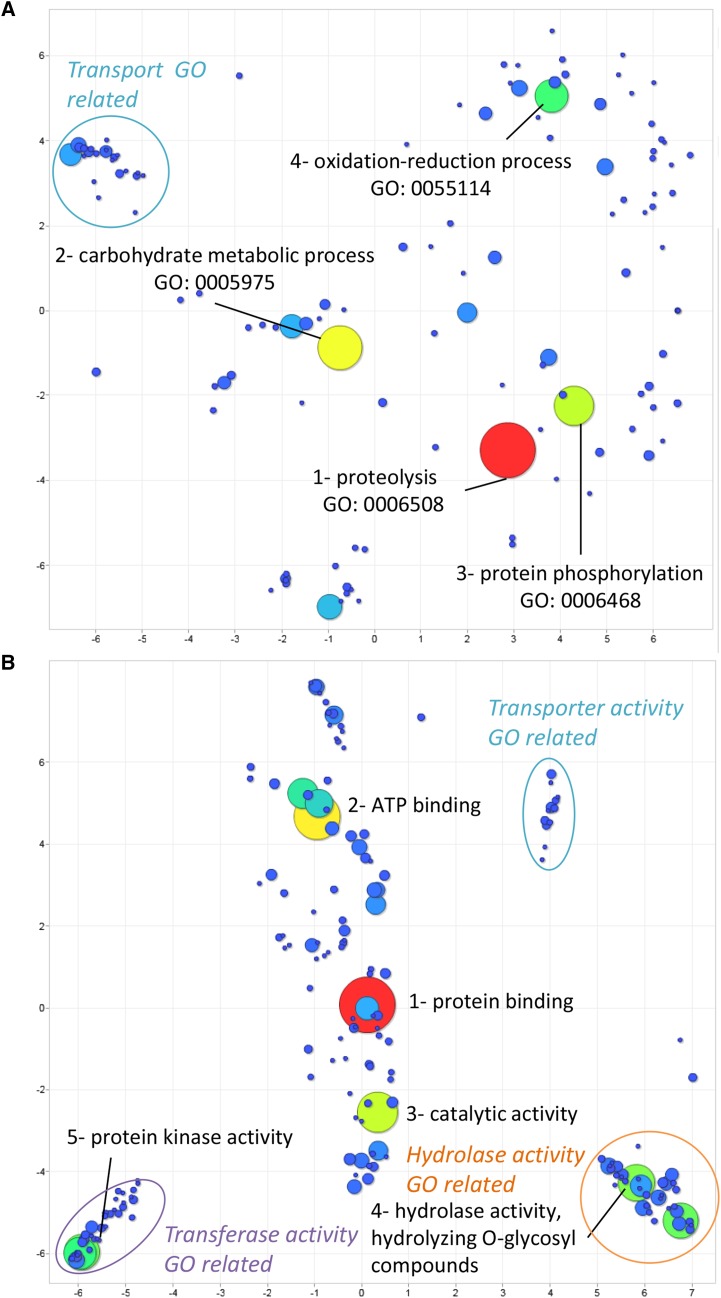
Biological Process and Molecular Function GO enrichment of the secretome.Putative secreted protein functional enrichment of GO terms. GO terms were summarized using REVIGO. Filled circle color and size indicate the GO occurrence in the *P. oligandrum* predicted secretome. Clear/unfilled circles indicate semantically similar GO terms group. (A) Biological Process GO terms enrichment overview and (B) Molecular Function GO terms enrichment overview.

Using SignalP v5 (Almagro Armenteros *et al.* 2019) 1,617 secreted proteins were predicted (Table S2). RNAseq data showed that 1,484 (91.8% of the putative secreted proteins) are expressed (Table S2), 1,020 of them have an InterPro annotation including 34 DUFs (Table S2) and 258 of them have a KEGG hit (Table S2. These putative secreted protein-coding genes were also functionally annotated with Gene Ontology (GO) terms using the InterPro annotation to understand the major biological and molecular role of the predicted secretome. Of the 1,617 secreted proteins, 699 (43.2%) have a GO term annotation (Table S2). To get an overview of the secreted protein major functions, GO terms annotation were summarized using REVIGO (Supek *et al.* 2011). Enriched terms were summarized by GO terms semantic similarities in a two dimensional scatterplots for each Gene ontology domains (cellular component, molecular function and biological process). Predicted proteins may have more than one GO term, for each gene all the GO classification was included in the REVIGO analysis. Circle size and color indicate the number of occurrence of the GO term in the secreted protein annotation list. Biological process GO terms scatterplot revealed strong representation of proteolysis (GO: 0006508), protein phosphorylation (GO: 0006468), carbohydrate metabolic process (GO: 0005975) and oxidation-reduction process (GO: 0055114) with two aggregation of semantic points on transport, amino-acids and carbohydrate biosynthesis related Gene Onthologies (Figure 2A)(Table S3). Molecular function GO terms scatterplot revealed stronger occurrence of GO terms corresponding to protein binding (GO: 0005515), ATP binding (GO: 0005524), protein kinase activity (GO: 0004672) and catalytic activity (GO: 0003824) with three aggregation of semantic points on transferase activity GO related, transporter activity GO related and hydrolase activity GO related (Figure 2B)(Table S4). These results suggest that the mycoparasitism pathogenicity of *P. oligandrum* ATCC 38472 is probably based on protein degradation and on polysaccharide degradation activities. Consistent with this idea, a total of 490 proteins contained a CAZy domain (Cantarel *et al.* 2009) (Carbohydrate Active Domain) predicted by dbCAN2 (Zhang *et al.* 2018) using the HMMER method. We annotated an extensive repertoire of 188 hydrolases of cell wall polymers derived from plants or fungi. Most of these hydrolases were predicted to be secreted (14 out of188) supporting the hypothesis that they directly interact with host cell wall and contribute to pathogenicity and mycoparasitism (Table S5). *Pythium oligandrum* ATCC38472 Glycoside Hydrolases (GHs) number has been compared to several phytopathogenic *Pythium* (Pyap, *P. aphanidermatum*; Pyar, *P. arrhenomanes*; Pyir, *P. irregulare*; Pyiw, *P. iwayamai*; Pyuu, *P. ultimum* var. *ultimum*; Pyus, *P. ultimum* var. *sporangiiferum*; Pyve, *P. vexans*) ones using available CAZyomes previously annotated with HMMER (Zerillo *et al.* 2013)(Table S6).

This analysis showed an enrichment of enzymes degrading fungal wall components such as β-1,3-glucanase (GH17, GH55). Among enzymes absent in phytopathogenic *Pythium and* related to fungal cell wall degradation, we found 13 GH19 chitinase, 3 GH46 chitosanase, 1 GH131 and 1 GH152 as β-1,3-glucanase that are specific to *P. oligandrum*. Moreover, a lack of GH16 xyloglucanase and GH28 polygalacturonase may have led to the loss of *P. oligandrum* ability to colonize efficiently plant tissues. In addition 156 secreted proteases (Supplementary Table 7), 303 Small Secreted Proteins (less than 300 amino acids with no functional annotation, (Gaulin *et al.* 2018) (Table S8) and 27 CRN-like proteins, known to participate in oomycetes pathogenicity (Amaro *et al.* 2017) were detected (Table S9). These data suggest that some of these secreted proteins may represent mycoparasitic specific functions (Table 2).

**Table 2:** Gene prediction and annotation summary

	Augustus prediction
Gene number	15,007
Busco on stramenopiles	232/234 (99.1%)
InterProScan domain	10,657
Secreted proteins (SignalP)	1,617
InterProScan + SignalP	1,020
CaZy domain	490
Putative CRNs	27
Putative SSPs	303

## Conclusion

Here, we present the first long-read genome sequence of *P. oligandrum*, thus providing a reference sequence for assembly and genomic analysis within the Pythiale genus. A large amount of the predicted genes are supported by transcriptomic data (95.25%). We focused on *P. oligandrum* ATCC 38472 secreted proteins to decipher potential mycoparasitism mechanisms and found that 10.7% of the total proteome possess a signal peptide. CAZyome annotation revealed enrichment and specific glycoside hydrolases related to fungal cell wall degradation, likely related to mycoparasitism. Oomycete pathogenicity toward diverse plant and animal host relies on such proteins to enter host cells and manipulate intracellular functions. Thus we will leverage this data to perform functional studies of *P. oligandrum* mycoparasitism.
